# P-1669. Prevalence of and Factors Associated with Long COVID among US Adults: A Nationwide Survey

**DOI:** 10.1093/ofid/ofaf695.1843

**Published:** 2026-01-11

**Authors:** Juanjuan Shi, Rui Lu, Yan Tian, Fengping Wu, Xiaozhen Geng, Song Zhai, Xiaoli Jia, Shuangsuo Dang, Wenjun Wang

**Affiliations:** Second Hospital of Xi'an Jiaotong University, Xi'an, Shaanxi, China; Second Hospital of Xi'an Jiaotong University, Xi'an, Shaanxi, China; Second Hospital of Xi'an Jiaotong University, Xi'an, Shaanxi, China; Second Hospital of Xi'an Jiaotong University, Xi'an, Shaanxi, China; Second Hospital of Xi'an Jiaotong University, Xi'an, Shaanxi, China; Second Hospital of Xi'an Jiaotong University, Xi'an, Shaanxi, China; Second Hospital of Xi'an Jiaotong University, Xi'an, Shaanxi, China; Second Hospital of Xi'an Jiaotong University, Xi'an, Shaanxi, China; Second Hospital of Xi'an Jiaotong University, Xi'an, Shaanxi, China

## Abstract

**Background:**

Nationwide data on long COVID prevalence and associated factors among US adults are scarce. This study aimed to determine long COVID prevalence and factors associated with among US adults using nationally representative data.

**Figure:**
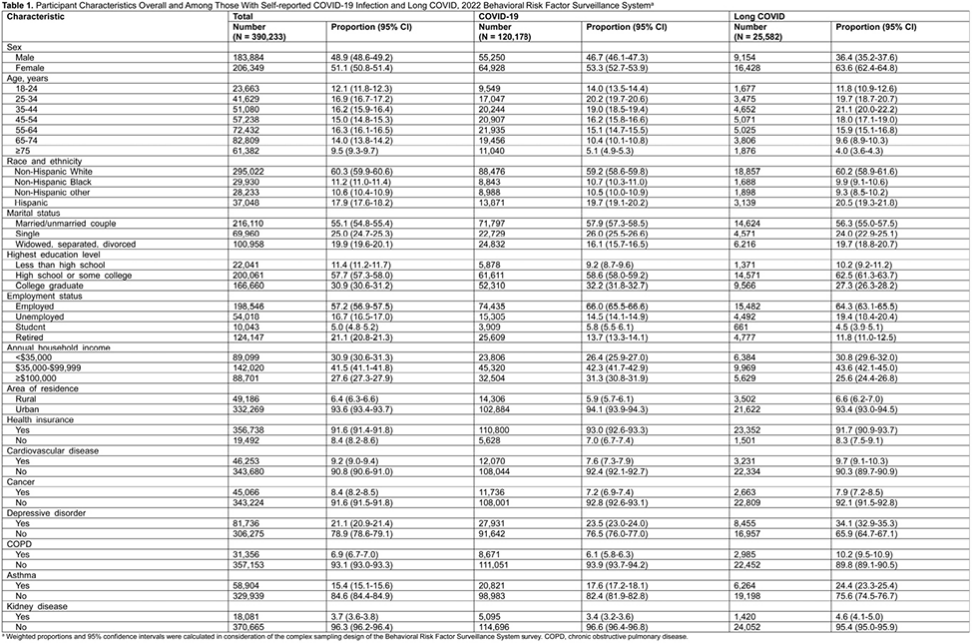

**Figure:**
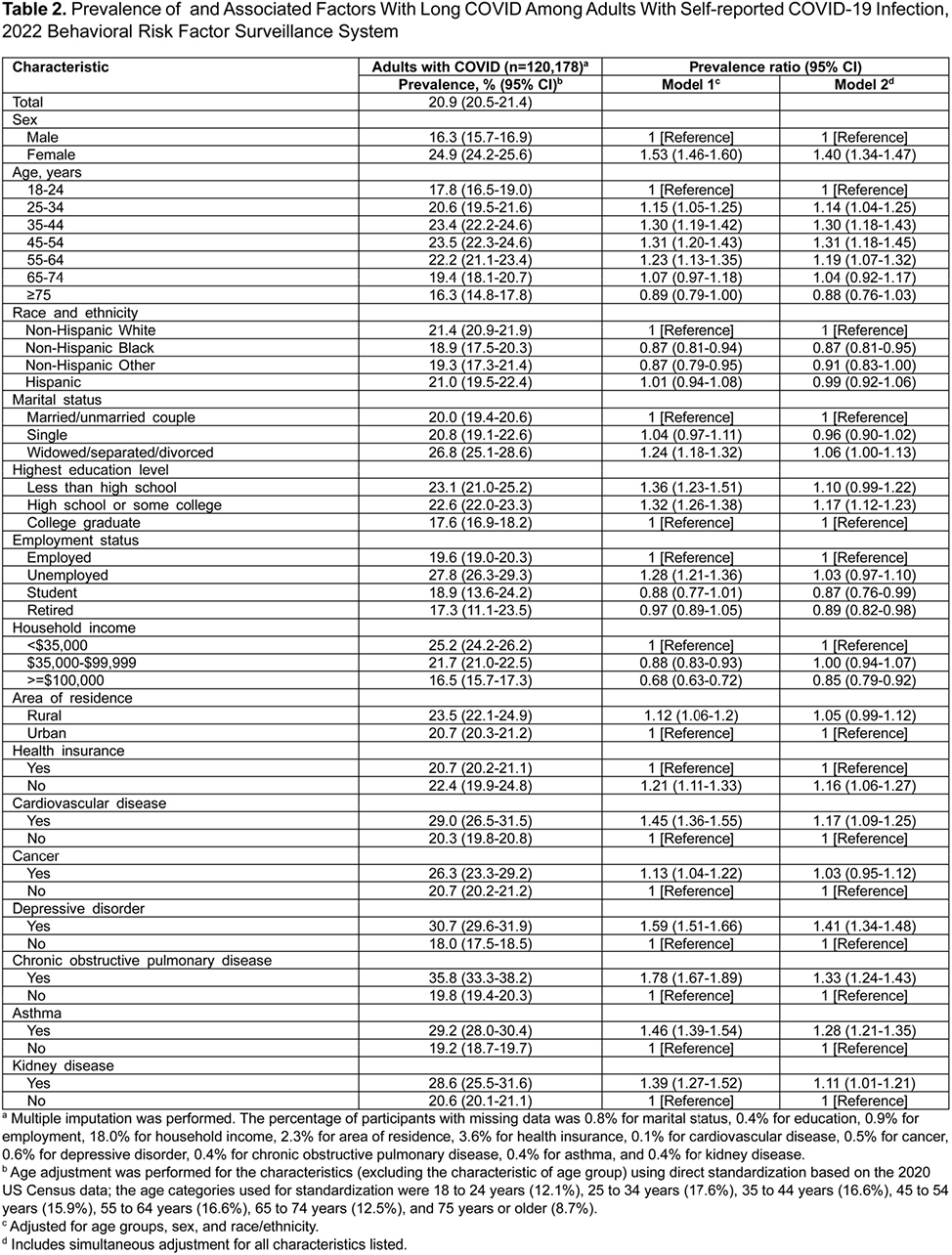

**Methods:**

This cross-sectional analysis utilized data from 2022 Behavioral Risk Factor Surveillance System survey, a nationally representative telephone survey conducted among noninstitutionalized adults aged ≥18 years residing in the United States.

**Figure:**
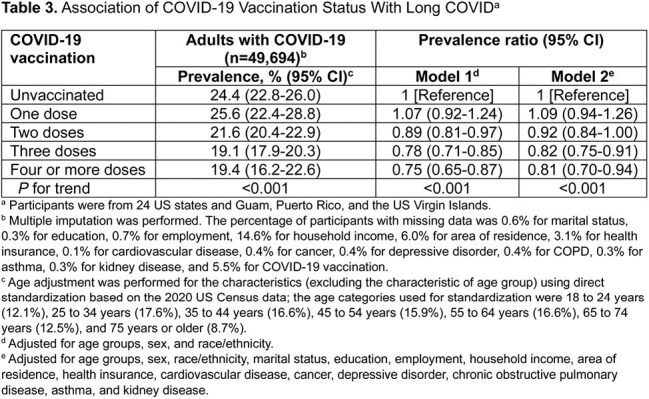

**Figure 1.**
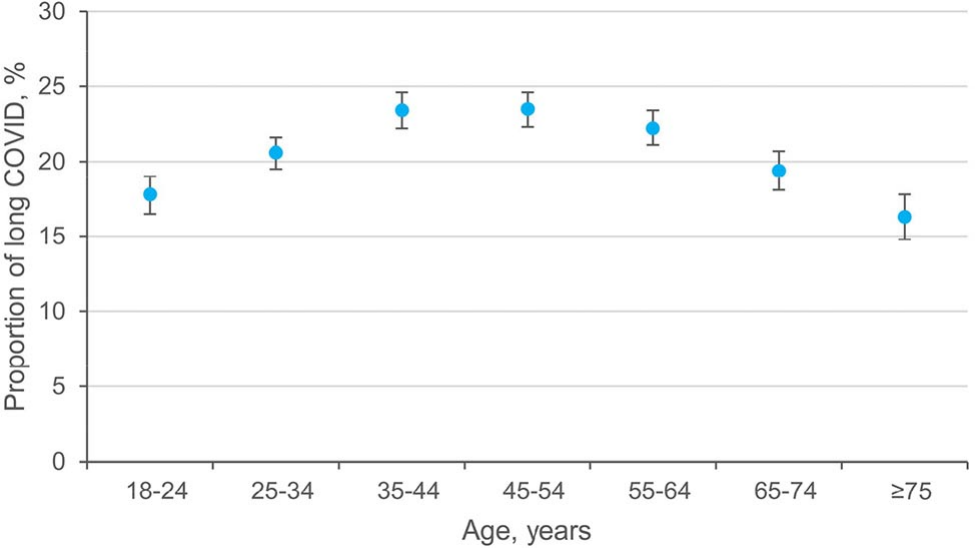
The relationship between age and long COVID reporting among US adults with self-reported COVID-19 infection. Data are presented as percentages and 95% confidence intervals.

**Results:**

Among 390,233 participants, 120,178 reported COVID-19, with 25,582 experiencing long COVID. Age-adjusted prevalence of self-reported COVID-19 and long COVID were estimated at 34.1% (95% CI, 33.7%-34.4%) and 7.2% (95% CI, 7.0%-7.4%) as of 2022, respectively. Among adults reporting COVID-19, 20.9% (95% CI, 20.5%-21.4%) had ever experienced long COVID. An inverted U-shaped association was observed between long COVID risk and age. Long COVID was more prevalent among women (adjusted prevalence ratio [aPR], 1.40 [95% CI, 1.34-1.47]), individuals without a spouse (aPR, 1.06 [95% CI, 1.00-1.13]), uninsured (aPR, 1.16 [95% CI, 1.06-1.27]), and those with a high school education (aPR, 1.17 [95% CI, 1.12-1.23]), cardiovascular disease (aPR, 1.17 [95% CI, 1.09-1.25]), depressive disorder (aPR, 1.41 [95% CI, 1.34-1.48]), chronic obstructive pulmonary disease (aPR, 1.33 [95% CI, 1.24-1.43]), asthma (aPR, 1.28 [95% CI, 1.21-1.35]), and kidney disease (aPR, 1.11 [95% CI, 1.01-1.21]). Long COVID was less prevalent among non-Hispanic Black (aPR, 0.87 [95% CI, 0.81-0.95]), students (aPR, 0.87 [95% CI, 0.76-0.99]) or retired individuals (aPR, 0.89 [95% CI, 0.82-0.98]), and those with household incomes ≥$100,000 (aPR, 0.85 [95% CI, 0.79-0.92]). Additionally, long COVID was less prevalent among those who received vaccination of two doses (aPR, 0.92 [95% CI, 0.84-1.00]), three doses (aPR, 0.82 [95% CI, 0.75-0.91]), and four or more doses (aPR, 0.81 [95% CI, 0.70-0.94]), compared with unvaccinated adults.

**Conclusion:**

Approximately 7.2% of US adults had experienced long COVID as of 2022. Female gender, middle age, White ethnicity, lower socioeconomic status, and various comorbidities are associated with higher long COVID rates, while vaccination with two or more doses is linked to lower rates.

**Disclosures:**

All Authors: No reported disclosures

